# Supplementary material for: Complexome profiling of the Chlamydomonas psb28 mutant reveals TEF5 as an early PSII assembly factor
Source: Plant Cell. 2025 Mar 17;37(6):koaf055. doi: 10.1093/plcell/koaf055 (PMC12164590; doi:10.1093/plcell/koaf055)
Supplement: koaf055_Supplementary_Data [file koaf055_supplementary_data.zip › psb28_interactive.zip!/plots/intensity/qualityPlots/peptides/_Cre11.g468353.t1.1.html]

### Cre11.g468353.t1.1

Trivial name:   
SeqCoverage [median]: 1.30  
Mass: 135.06 kDa  
  
 

| Sequence | MissedCleavage | Mass [Da] | wtIntensity | muIntensity | Score |
| --- | --- | --- | --- | --- | --- |
| DGFAAPTKK | 1 | 933.49 | 7891.18 | 7318.08 | 91.85 |
| IVKSMPRLR | 2 | 1098.67 | 0.00 | 1811.66 | 64.71 |
|  |  |  | 7891.2 | 9129.7 |  |
